# Astrocytes Derived from Familial and Sporadic Alzheimer’s Disease iPSCs Show Altered Calcium Signaling and Respond Differently to Misfolded Protein Tau

**DOI:** 10.3390/cells11091429

**Published:** 2022-04-22

**Authors:** Veronika Brezovakova, Eva Sykova, Santosh Jadhav

**Affiliations:** Institute of Neuroimmunology, Centre of Excellence for Alzheimer’s Disease and Related Disorders, Slovak Academy of Sciences, Dubravska 9, 845 10 Bratislava, Slovakia; veronika.brezovakova@savba.sk (V.B.); sykovae@gmail.com (E.S.)

**Keywords:** astrocytes, Alzheimer’s disease, iPSCs, tau, matrix metalloproteinases

## Abstract

Astrocytes regulate important functions in the brain, and their dysregulation has been linked to the etiology of neurodegenerative diseases, such as Alzheimer’s disease (AD). The role of astroglia in human AD remains enigmatic, owing to the limitations of animal models, which, while recreating some pathological aspects of the disease, do not fully mirror its course. In addition, the recognition of major structural and functional differences between human and mouse astrocytes has also prompted research into human glial cells. In the current study, astrocytes were generated using human iPSCs from patients with sporadic Alzheimer’s disease (sAD), familial Alzheimer’s disease (fAD) and non-demented controls (NDC). All clones gained astrocyte-specific morphological and proteomic characteristics upon in vitro differentiation, without considerable inter-clonal variances. In comparison to NDC, AD astrocytes displayed aberrant calcium dynamics in response to glutamate. When exposed to monomeric and aggregated tau, AD astrocytes demonstrated hypertrophy and elevated GFAP expression, differential expression of select signaling and receptor proteins, and the enhanced production of metalloproteinases (MMPs). Moreover, astrocytic secretomes were able to degrade tau in both monomeric and pathologically aggregated forms, which was mediated by MMP-2 and -9. The capacity to neutralize tau varied considerably between clones, with fAD astrocytes having the lowest degradability relative to sAD and healthy astrocytes. Importantly, when compared to aggregated tau alone, astrocytic secretome pretreatment of tau differentially reduced its detrimental effects on neurons. Our results show crucial differences in sporadic and familial AD astrocytes and suggests that these cells may play distinctive roles in the pathogenesis of early and late onset Alzheimer’s disease.

## 1. Introduction

Alzheimer’s disease (AD), the most common cause of dementia in the elderly, is characterized by the presence of tau tangles, amyloid plaques and neuroinflammation. Based on its etiology, the disease is defined by two classes, familial AD (fAD), with mutations in key proteins involved in AD, and sporadic (sAD) (no dominant mutations); however, life style, genetic susceptibility (such as ApoE, TREM2, etc.), and environmental and immune factors are considered as contributing factors [1]. Genome-wide association studies also suggest a number of genetic foci as risk factors in sporadic AD [2,3]. In terms of disease progression, fAD manifests early and shows a robust pathology and neurodegeneration when compared to sporadic forms of AD, which manifest later in the elderly and usually have a slower time course.

Evidence points to the role of astrocytes in pathogenesis of AD [4,5]. In a healthy brain, astrocytes play vital functions such as in glutamate release and uptake, K^+^, pH and water homeostasis [6], synapse formation, pruning and activity [7], and the integrity of the blood–brain barrier [8]. In AD, reactive astrogliosis and the hypertrophy of astrocytes in regions surrounding amyloid beta plaques (Aβ) and neurofibrillary tangles (NFTs) are observed [9,10]. It was also shown that astrocytes participate in the clearance of amyloid beta [11,12], via matrix metalloproteinases (MMPs). Moreover, in a transgenic rodent model of AD, the astrocytes in proximity to amyloid plaques were highly reactive to MMP-2 and MMP-9 [11,12]. In addition, the co-localization of MMP-2 with neurofibrillary tangles and dystrophic neurites was observed in the early stages of AD, highlighting a crucial role of the MMPs in AD pathogenesis [13].

Tau protein is released into the extracellular space between neurons which contributes to the propagation of tau pathology [14]. Consequently, the effect of tau and its uptake by microglia, the resident macrophages of the CNS, has been widely studied [15,16]. In contrary, very limited information on the role of astroglia in regard to tau pathology is available. It is shown that astrocytes can uptake tau [17,18], and astroglial tau accumulation is observed in several tauopathies, broadly known as aging-related tau astrogliopathy [19,20]. As of now, no study has investigated the uptake of tau and its effect on AD-derived astrocytes [21]. It is yet unknown at what rate astrocytes can degrade tau, and whether AD-derived astrocytes can uptake/degrade tau.

On the other hand, our current understanding of AD stems from models that harbor mutations such as in APP or PSEN [22], which develop an extensive amyloid pathology, but minimal tau associated pathology. Likewise, models established on the overexpression of tau or with tau mutations fail to develop any amyloid pathology [23,24]. In addition, our current knowledge on the role of astrocytes in diseases is built on postmortem studies or rodent models [25,26]. Seemingly, human astrocytes are more diverse than rodent astrocytes, and respond differently to stimuli [27,28]. Therefore, the lack of models to faithfully study AD and the contribution of astrocytes in AD pathogenesis is a major limitation in the development of promising therapeutics for this devastating neurodegenerative disease.

In recent years, induced pluripotent stem cells (iPSCs) have been widely used as a platform to model a number of diseases, including AD [29]. It is argued that iPSC-derived neuronal stem cells (NSCs) can better reflect the pathogenesis of AD, when compared to conventional transgenic models, and can serve as a tool to identify genetic and environmental risk factors in the onset of sporadic AD [29,30]. Several studies have utilized human-derived iPSC as models to investigate AD in both familial and sporadic subjects [31,32,33,34,35]. In this study, we compared iPSC-derived astrocytes from healthy controls and sporadic and familial AD subjects, and proceeded to elucidate the effect of tau proteins on these cells. Our research, for the first time, investigated the impact of tau protein on iPSC-derived astrocytes from sporadic and familial Alzheimer’s disease, and provides insights on the differential response of these cells when compared to healthy control iPSC-derived astrocytes.

## 2. Materials and Methods

### 2.1. Culture and Differentiation of Neural Stem Cells

Human iPSC-derived neural stem cell (NSC) clones from non-demented control (NDC), sporadic (sAD) and familial AD (PSEN-1 mutation) (fAD; Supplementary Table S1) subjects were used in this study (Biotalentum Ltd., Gödöllő, Hungary). Three independently generated clones for each group were used. The generation and characterization of the clones has been previously described [36,37,38]. All clones tested negative for mycoplasma. Initial experiments were performed using all 3 clones per line for consistency. Subsequent experiments were performed using one clone per line in every experiment, and the same clone was used in all experimental conditions and presented in this study. The work was approved by the ethical committee of Institute of Neuroimmunology, Slovak Academy of Sciences.

The NSCs were plated on Poly-L-ornithine/Laminin (pLO/L; Sigma, Bratislava, Slovakia) coated 6-well plates (Thermo-Scientific, Bratislava, Slovakia) and propagated in neuronal maintaining medium (NMM), consisting of Neurobasal and DMEM/F12 media 1:1 supplemented with Glutamax, 1% (Gibco, Bratislava, Slovakia) N2 (Gibco), 1% B27 (Gibco), 1% NEAA (Gibco), 1% Penicillin/Streptomycin (Gibco) with 10 ng/mL bFGF and 10 ng/mL of EGF (Peprotech, Cranbury, NJ, USA). NMM was changed every other day until 95% confluence, upon which cells were passaged and expanded/cryo-banked (Supplementary Figure S1a). Highly pure NSCs, as assessed by markers SOX-2, PAX-6 and Nestin, were used for terminal differentiation (Supplementary Figure S1b).

#### 2.1.1. Differentiation of Astrocytes

For differentiation into astrocytes, cells were plated on pLO/L coated 6-well plates and NMM was switched to NSC differentiation medium Astrocyte lineage (Cell Applications, Inc., San Diego, CA, USA). Media (50%) were changed daily, and cells were passaged at 90-95% confluence. After a week, cells were switched to Matrigel-coated plates (#354277, Corning, Bedford, MA, USA) with a media change every other day for ~2 weeks and passaged using Cell Recovery Solution (Corning, Bedford, MA, USA). For final maturation, astrocytes were seeded in a liquid Matrigel: media mixture (1:10) at a final density of 2 × 10^6^ cells/mL for 12-well-format or 3–5 × 10^4^ cells for 8-well Lab-Tek II Chamber Slide (Thermo-Scientific, Bratislava, Slovakia) unless stated otherwise, solidified by 35 min incubation at 37 °C, 5% CO_2_ (3D layer) and cultured in Astrocyte Growth Media [39] (Cell applications, Inc., San Diego, CA, USA) for up to 30 days.

#### 2.1.2. Differentiation of Neurons

For differentiation into neurons, cells were plated on Matrigel-coated 12-well plates or 8-well Lab-Tek slides with a seeding density of 1.5–3 × 10^4^ cells/cm^2^. NMM media were switched to Neuronal Medium (#05790, Stemcell Technologies, Cologne, Germany), supplemented with 1% N2 Supplement-A+ 2% SM1 Neuronal Supplement (# 05711, Stemcell Technologies), 20 ng/mL GDNF, 20 ng/mL BDNF, 1 mM Dibutyryl-cAMP, 200 nM (Gibco), and Ascorbic acid (Peprotech). Half media change was performed every 48 h and cells were cultured till day 30. For subset of experiments, neurons were supplemented with astrocyte-conditioned media (ACM) (300 µg of protein) from NDC, sAD or fAD astrocytes, and cultured for additional 5 days with ACM renewal every 48 h.

### 2.2. Sample Preparation

For the generation of astrocyte-conditioned media (ACM), cells upon reaching 90% confluence were switched to media without supplements (#05790, Stemcell Technologies) and sub-cultured in a humidified chamber at 37 °C with 5% CO_2_. After 48 h, ACMs were harvested, centrifuged at 1000× *g*/10min to eliminate cell debris and concentrated using 3 kDa molecular weight cutoff filter (Millipore, Burlington, MA, USA). Protein concentration was estimated by Bradford assay (Bio-rad laboratories Inc., CA, USA) and ACMs aliquots were stored at −80 °C until use.

For the cell lysate, cells were homogenized in buffer containing 20 mM Tris pH 7.4, 150 mM NaCl, 1 mM EDTA, 2 mM DTT and 0.5% Triton X-100 with protease inhibitors (Roche Diagnostics GmbH, Mannheim, Germany). The lysates were clarified at 20,000× *g* for 20 min at 4 °C and the resulting supernatant was collected and stored at −80 °C until use. Protein concentrations of samples were estimated using the Bradford method.

### 2.3. Immunocytochemistry

Cells were fixed with 4% paraformaldehyde for 20 min and washed. Unspecific antibody binding was blocked using 5% normal goat serum followed by incubation with primary antibodies overnight at 4 °C. After washing, the cells were incubated with their respective Alexa conjugated secondary antibodies (Thermo-Scientific, Bratislava, Slovakia) for 1 h at room temperature in dark. The slides were mounted with coverslips using Fluorshield^TM^ with DAPI to visualize nuclei (Sigma). The following antibodies were used: GFAP (1:500, ab10062; Abcam, Bratislava, Slovakia), ACSA-1 (GLAST) (1:500; #130-095-822; Miltenyi biotech), ALDH1L1 (1:500; MABN495; Bratislava, Millipore), S100β (1:500; ab41548; Abcam), β-tubulin III (Tuj1) (1:500; ab78078; Abcam), MAP-2 (1:500; ab5392; Abcam), Synapsin 1 (1:1000; ab64581; Abcam), PAX-6 (1:1000; ab195045; Abcam), SOX-2 (1:500; ab92494; Abcam), and Nestin (1:500; ab22035; Abcam).

### 2.4. Calcium Imaging

Cells were first loaded with fluo-4 AM (1 μM; Thermo-Scientific, Bratislava, Slovakia) at 37 °C for 60 min in dark, as per the manufacturer’s instructions. Cells were washed with probe-free medium and maintained at room temperature in the dark until data acquisition. Cells were challenged with 10 μM Glutamate (neurons) or 100 μM Glutamate (astrocytes), as previously described [40]. Cells were excited at 488 nm wavelength and images were acquired at 520 nm. Fluorescent signals were recorded using LSM 710 confocal microscope with Zen software system (Carl-Zeiss). Time-lapse (10 s frame) movies of calcium flux are provided as Supplementary file. To assess difference in calcium uptake of astrocytes the signals were obtained from five randomly chosen areas per well, and analysis was performed using CALIMA [41]. For quantification, the Ca^2+^-positive cells from 5 random fields were calculated by blind observer using Fiji software. The duration of calcium transients was measured using CaSiAn [42].

### 2.5. Tau Protein Labeling and In Vitro Aggregation and Uptake

The expression and purification of truncated tau was performed, as previously described [43]. The labeling of recombinant tau was performed using Alexa Fluor™ 546 Protein Labeling Kit (#A10237; Thermo-Scientific, Bratislava, Slovakia), according to the manufacturer’s instructions. The in vitro aggregation of recombinant truncated tau protein (aa151-391, based on longest tau isoform tau 40) was performed using heparin (Sigma) as an inducer at a final concentration of 25 μM in 1× PBS (137 mM NaCl, 2.7 mM KCl, 10 mM Na_2_HPO_4_, 2 mM KH_2_PO_4_, pH 7.4) overnight at 37 °C, as previously described and characterized [44]. Post incubation, the sample was centrifuged at 100,000× *g* for 1h at room temperature, and the pellet was re-suspended in 1× PBS, sonicated for 5 s at 20% power output using an MS72 probe of a Bandello Sonopuls Sonifier (Bandelin, Berlin, Germany), and stored as 1 μM aliquots at −80 °C. The oligomerization of the tau protein was verified by immunoblotting and electron microscopy (Supplementary Figure S2a,b). For the examination of tau uptake by astrocytes, cells were plated on 8-well Lab-Tek slides (25,000 cells/well), incubated with labeled monomeric (4 µM)/aggregated tau (1 µM), and washed and examined using LSM 710 confocal microscope (Carl-Zeiss, Jena, Germany). Images were obtained using the Zen software system (Carl-Zeiss, Jena, Germany) and fluorescent intensities were measured using Fiji (ImageJ 2.0.0). For the compartmentalization study, SiR-lysosome kit—Live cell lysosome probe (Spirochrome, Stein am Rhein, Switzerland) was used according to the manufacturer’s instructions.

### 2.6. Immunoblotting

Protein samples were subjected to SDS-PAGE separation and transferred to a nitrocellulose membrane. After blocking using 5% milk or 5% BSA for 30 min, the membranes were incubated with primary antibodies for 2h at room temperature or overnight at 4 °C. Membranes were incubated with HRP-conjugated secondary antibodies for 1 h at RT (Dako, Glostrup, Denmark), and developed using an enhanced chemiluminescence Western blotting detection kit (Thermo-Scientific, Bratislava, Slovakia) on Image Reader LAS-3000 (FUJI Photo Film Co., Ltd., Tokyo, Japan). For the comparison of phospho-protein to total protein levels, the membranes were stripped and incubated with total protein antibodies. The intensity of the bands was assessed semi-quantitatively using AIDA (AIDA Biopackage Raytest, Straubenhardt, Germany). The levels of proteins were normalized to total actin levels, and for phospho-proteins with respective total protein levels. The following antibodies were used: DC25 (1:1*; Axon Neuroscience, Bratislava, Slovakia; *supernatant from hybridoma cells), GFAP (1:1000; ab7260; Abcam), Connexin-43 (1:2000; ab11370; Abcam), Aquaporin-4 (1:1000; AB3594; Millipore), EAAT-2 (1:1000; AB1783; Millipore), Phospho-ERK1/2 (1:2000; 28733-1-AP; Proteintech), Total-ERK1/2 (1:2000; 16443-1-AP; Proteintech), Phospho-PKCα/β II (1:1000; #9375S; Cell Signaling Technology), Total-PKCα/β II (1:1000; #2056S; Cell Signaling Technology), MMP-9 (1:1000; #819701; Biolegend), MMP-2 (1:1000; #679902; Biolegend; Amsterdam, Netherlands), GluN1 (1:1000; #114011; SYSY), GAP-43 (1:1000; NBP1-71668; Novus biologicals), Drebrin (1:2000; ab178408; Abcam), and Actin (1:2500; ab76548; Abcam).

### 2.7. Electron Microscopy

The aggregation of tau was verified using electron microscopy. A drop of tau oligomer solution was placed on carbon-coated 400 mesh copper grids (Christine Gröpl, Tulln an der Donau, Austria) for 5 min. The grids were briefly washed with water and negatively stained with 2% uranyl acetate for 1 min (Sigma). After brief rinsing in water and drying, the grids were examined using an FEI Morgagni 268 electron microscope (FEI Czech Republic s.r.o.; Brno, Czech Republic).

### 2.8. In-Gel Zymography

Fifty micrograms of ACM were subjected to non-reducing SDS-PAGE separation with resolving gel containing 0.2% gelatin. After separation, the gels were washed twice (30 min each) with wash buffer containing 2.5% triton X-100, 50 mM Tris-HCl pH 7.5, 5 mM CaCl_2_ and 1 µM ZnCl_2_ with gentle agitation. Then, the gels were incubated overnight in incubation buffer containing 1% triton X-100, 50 mM Tris-HCl pH 7.5, 5 mM CaCl_2_ and 1 µM ZnCl_2_ at 37 °C. After a brief wash, gels were stained with Coomassie brilliant blue and destained until clear bands were obtained. The gels were scanned and the intensity of the bands was assessed semi-quantitatively using AIDA.

### 2.9. Tau Degradation and Inhibition Assays

Fifty micrograms of ACM (based on Bradford assay) was incubated with either 4 µM of recombinant monomeric tau 2N4R or 1 µM of aggregated tau aa151-391 in the presence or absence of MMP inhibitors for 6 h and 24 h at 37 °C (50 µM of MMP-9 Inhibitor I (#444278; Millipore, Bratislava, Slovakia), 17 µM of MMP-2 Inhibitor II (#444244; Millipore) or 10 µM universal MMP inhibitor EDTA (Sigma)). The final reaction volume was adjusted equally using sterile 1× PBS. The reaction was stopped by the addition of 2× Laemmli sample loading buffer followed by boiling. The samples were subjected to SDS-PAGE separation and immunoblotting analysis using Pan-tau antibody DC25. The data were normalized to the percentage band intensity of load (run in same gel) to band intensity postdegradation/-inhibition.

### 2.10. Viability and Cytotoxicity Assay

A cytotoxicity assay was performed using the Toxilight^TM^ Bioassay kit (#LT07-217; Lonza, Basel, Switzerland) according to the manufacturer’s instructions. Briefly, neurons differentiated from iPSCs were supplemented with ACM post aggregated tau pretreatment or aggregated tau alone, for 24 h. The media were collected and incubated with AK detection reagent for 5 min at room temperature, and luminescence intensity was measured as 1 sec integrated reading of appropriate wells using Fluorscan™ Microplate reader (Thermo-Scientific, Bratislava, Slovakia). For general cell viability, a Non-Radioactive Cell Proliferation Assay (MTT) assay was performed as per the manufacturer’s instructions (#G4000; Promega, Madison, WI, USA) and expressed as the percentage of live cells.

### 2.11. Statistical Analysis

All the experiments were repeated at least three times. Statistical analyses were performed with Prism GraphPad Software Version 7 (Graph Pad Software Inc., San Diego, CA, USA). All data sets are expressed as a mean ± standard deviation (SD). One-way ANOVA followed by Tukey’s test for multiple comparisons was used for statistical analysis. The criterion for statistical significance was *p* < 0.05, and statistical differences are indicated as * *p* < 0.05, ** *p* < 0.01, and *** *p* < 0.001. All graphs were generated using GraphPad.

## 3. Results

### 3.1. Generation and Characterization of Astrocytes from Human iPSC-Derived Neuronal Stem Cells

**Figure 1:**
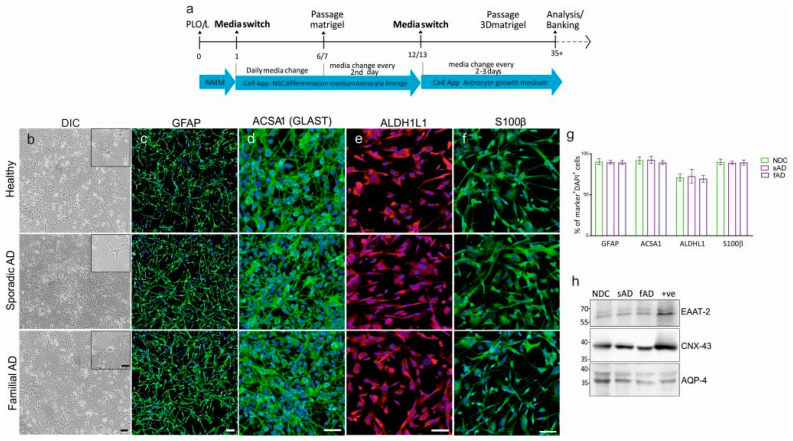
Differentiation of astrocytes from iPSC-derived neuronal stem cells from non-demented control, sporadic and familial Alzheimer’s disease. (**a**) Scheme showing differentiation of astrocytes from iPSC-derived NSCs. (**b**) Phase contrast images of astrocyte monolayer after 30 days of induction. Scale bar: 50 μm; inset: 20 μm. No prominent morphological difference in astrocytes was observed between the three groups. Representative images of markers for astrocytes differentiated from non-demented control, sporadic and familial iPSC-derived neuronal stem cells using antibodies against (**c**) GFAP, (**d**) ACSA 1, (**e**) ALDH1L1, and (**f**) S100β. DAPI was used for nuclear visualization. Scale bar: 50 μm. (**g**) Graph showing percentage of cells positive for individual markers in (**c**–**f**). One-way ANOVA followed by Tukey’s multiple comparisons tests. (**h**) Immunoblots of astrocyte cell lysates after differentiation using antibodies against astrocyte markers EAAT-2, Conexin-43 (CNX-43) and Aquaporin-4 (AQP-4). Human brain cell lysate was used as a positive control (+ve). Results are expressed as mean ± SEM, *n* = 3/group; *n* = 3 experimental repeats.

Astroglial differentiation was induced using defined human astrocyte (HA) differentiation medium and maintained in HA growth media from Cell applications, as detailed in the methods section (Figure 1a).

No evident changes in the differentiation potential among each clone (AD and NDC) was observed. The matured cells acquired a stellate morphology comparable between healthy controls and AD subjects. Independent of the subject status, derived astrocytes expressed similar levels of cardinal astrocytic markers glial fibrillary acidic protein (GFAP), glutamate aspartate transporter 1 (GLAST-1 or EAAT-1), aldehyde dehydrogenase 1 family member L1 (ALDH1L1), and S100 calcium-binding protein B (S100β) (Figure 1b–g), along with Excitatory amino acid transporter 2 (EAAT-2), water-channel AQP4, and gap junction protein Connexin-43 (CNX-43) (Figure 1h). To summarize, these results show the successful induction and generation of cells from different NSCs with morphological and molecular features typical of astrocytes in vitro.

**Figure 2:**
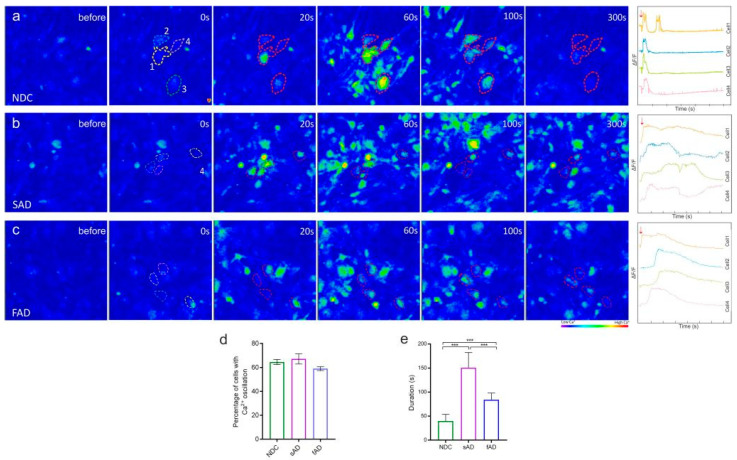
AD-derived astrocytes show increased response to glutamate stimulation in vitro. Representative field images of glutamate stimulated astrocytes from (**a**) NDC, (**b**) sAD, and (**c**) fAD. Representative images from calcium recordings of four individual cells (location of selected cells marked and numbered in respective fields). Changes in fluorescence intensity were measured using CALIMA software and indicated as ratio measured fluorescence intensity (F) to measured average baseline fluorescence intensity (F/F0) at time (s). Arrow indicates point of glutamate addition. Note the altered stimulation in astrocytes from sAD and fAD when compared to astrocytes from NDCs. (**d**) Graph showing percentage of calcium-positive cells post stimulation. (**e**) Graphs showing duration of calcium transients between the groups post glutamate stimulation. One-way ANOVA followed by Tukey’s multiple comparisons tests, *n* = 3/group; *n* = 3 experimental repeats, and *** *p* < 0.001.

Intracellular signaling in astrocytes is dependent on calcium fluctuations; therefore, we examined calcium influx using the Ca^2+^ indicator Fluo-4 AM between the lines. All derived astrocytes were able to propagate spontaneous intracellular Ca^2+^ transients (Supplementary Figure S2a–c). In addition, exogenous glutamate-induced (100 µm) calcium waves in all derived clones (Figure 2a–c; Supplementary Movies S1–S3). We did not observe any difference in the percentage of cells showing Ca^2+^ transients between the groups (Figure 2d); however, both familial and sporadic AD-derived astrocytes exhibited prolonged calcium transients (Figure 2b,e), which were more sustained in the sporadic AD astrocytes (Figure 2e) when compared to non-demented and fAD astrocytes (Figure 2a,e).

### 3.2. Tau Protein Induces Morphological Changes Associated with GFAP Upregulation Only in AD-Derived Astrocytes

**Figure 3:**
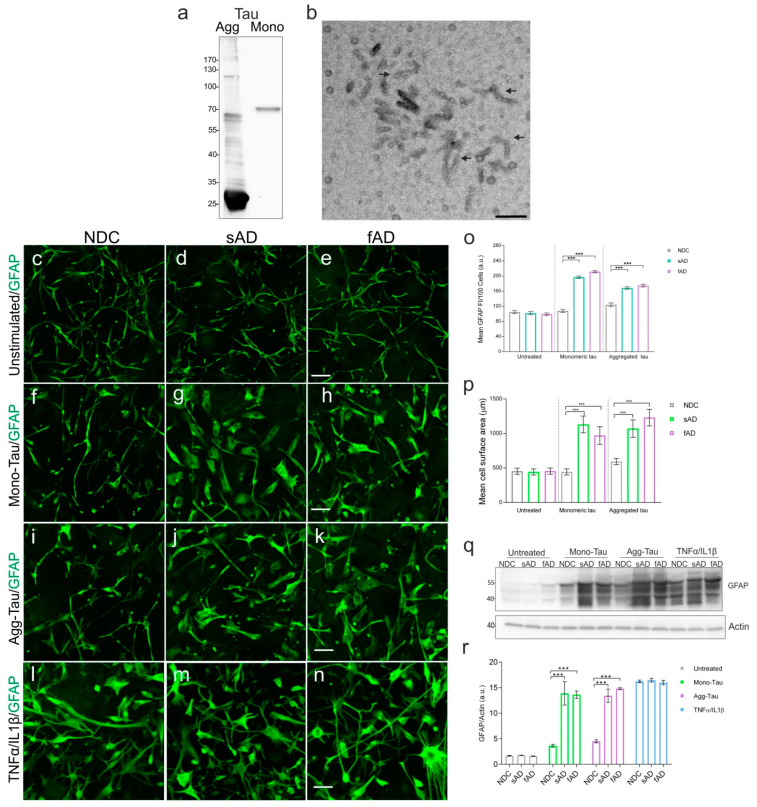
Astrocytes derived from AD behave differently to monomeric and aggregated tau in vitro. (**a**). Western blot showing aggregated and monomeric tau used in the study. (**b**). Electron microscope image of the aggregated tau preparation used in the study (arrows indicate tau fibrils and short filaments). Representative confocal images of GFAP-positive astrocytes. (**c**–**e**) Untreated, (**f**–**h**) astrocytes upon stimulation with monomeric and (**i**–**k**) aggregated tau. (**l**–**n**) Proinflammatory cytokine TNFα/IL1β cocktail was used as positive control to show astrocyte reactivity in all cases. Scale bar: 50 µm. (**o**) Graph showing difference in mean fluorescent intensities (±SD) of GFAP between the untreated iPSC-derived astrocytes and after exposure to monomeric and aggregated tau. (**p**) Graph showing mean surface area of cells pre- and poststimulation with monomeric and aggregated tau. (**q**) Western blot using anti-GFAP antibody using cell lysates from untreated, or after stimulation with monomeric, aggregated-tau or TNFα/IL1β cocktail. (**r**) Graph showing the levels of GFAP between the groups. One-way ANOVA followed by Tukey’s multiple comparisons tests, *n* = 3/group; *n* = 3 experimental repeats, and *** *p* < 0.001.

Astrocyte reactivity is characterized by morphological changes, resulting in cell hypertrophy accompanied by the upregulation of GFAP. We exposed astrocytes from NDC and ADs to 4 µM monomeric (MonoTau) or 1 µM of aggregated tau protein (AggTau) (Figure 3a,b). For morphological assessments, cells were exposed to non-labelled tau (Mono- or AggTau) and stained using anti-GFAP antibody.

Stellate morphology and baseline GFAP levels were similar in all untreated clones (Figure 3c–e). Upon exposure to tau (both Mono- and AggTau) the sAD (Figure 3g,j) and fAD (Figure 3h,k), cells showed hypertrophy with the deramification of the processes (Figure 3p), accompanied by increased levels (~2 fold) of GFAP protein (Figure 3o). No significant morphological changes occurred and a minute change in the expression of GFAP was observed in astrocytes from NDC in response to tau (Figure 3f,i). TNFα/IL1β treatment was used as the control to show that astrocytes from all three groups were reactive and respond to proinflammatory stimuli (Figure 3i–n). In addition, preblocking of Heparan Sulfate Proteoglycans (HSPG) with heparin (20 μg/mL for 3 h, which inhibits tau uptake) almost mitigated the effect of Mono- (Supplementary Figure S3g–i) and AggTau (Supplementary Figure S3j–l) on sAD and fAD astrocytes, when compared to heparin-untreated cells (Suplementary Figure S3a–f), suggesting a tau-specific effect on AD-derived astrocytes.

In order to confirm these results, we subjected astrocyte cell lysates to immunoblotting using anti-GFAP antibody. We observed a more than 2-fold increase in GFAP levels in Mono- and AggTau-treated sAD and fAD astrocytes (Figure 3q). Astrocytes from all groups showed a marked increase in GFAP expression after combined treatment with TNFα and IL1β (Figure 3q,r).

**Figure 4:**
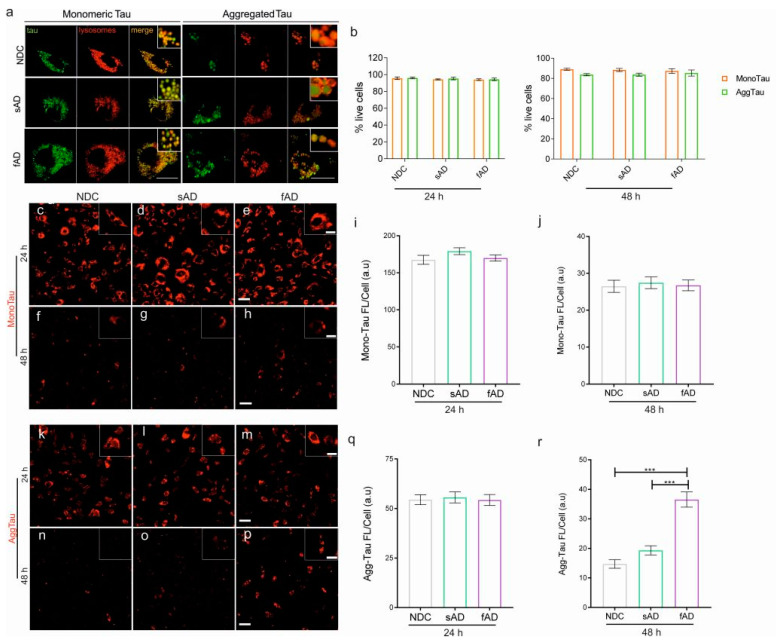
Astrocytes derived from fAD differently degrade monomeric and aggregated tau in vitro. (**a**) Bioimaging revealed the presence of monomeric and aggregated tau in the lysosomal compartment in all clones. Scale bar: 20 µm. (**b**) Graph showing percentage of viable astrocytes from NDC, sAD and fAD after exposure to monomeric or aggregated tau at 24 and 48 h. Bio-imaging of tau uptake by (**c**) NDC, (**d**) sAD and (**e**) fAD astrocytes 24 h after incubation with Alexa 546-conjugated monomeric tau. Representative live cell images of (**f**) NDC, (**g**) sAD and (**h**) fAD astrocytes at 48 h after removal of media containing monomeric tau (at 24 h). Graph showing mean fluorescence intensity of tau within the astrocytes at (**i**) 24 h and (**j**) 48 h, respectively. Bio-imaging of tau uptake by (**k**) NDC, (**l**) sAD and (**m**) fAD astrocytes 24 h after incubation with Alexa 546-conjugated aggregated tau. Representative live cell images of (**n**) NDC, (**o**) sAD and (**p**) fAD astrocytes at 48 h after removal of media containing aggregated tau (at 24 h). Note the drastic decrease in fluorescence intensity in the NDC and sAD astrocytes, and the accumulation of AggTau in astrocytes from fAD iPSCs at 48 h. Graph showing fluorescent intensity/cell of aggregated tau uptake at 24 h (**q**), and levels of aggregated tau at 48 h (**r**). One-way ANOVA followed by Tukey’s multiple comparisons tests was used for statistical analysis, (*n* = 3/group; *n* = 3 experimental repeats). *** *p* < 0.0001. Scale bar: 50 nm; inset: 20 nm.

In order to observe intracellular uptake, cells were subjected to bioimaging using fluorescently labeled tau. At 24 h, we observed that the tau protein was localized in the lysosomal compartment (Figure 4a), and all clones efficiently took up monomeric (Figure 4c–e) and aggregated tau species (Figure 4k–m). Exposure to monomeric or aggregated tau did not affect the viability of cells in all cases (Figure 4b). The mean fluorescence intensity threshold for MonoTau uptake was higher than that of AggTau (Figure 4i,q). To observe if the internalized tau is degraded by the astrocytes, the tau-containing media were replaced with media without tau at 24 h. After 48 h, we observed that the NDC, sAD and fAD astrocytes showed similar levels of degradation of monomeric tau (Figure 4f–h,j). We also observed that the astrocytes were able to degrade aggregated tau, but the fAD-derived astrocytes were less efficient in the degradation of aggregated tau, leading to more than 2-fold accumulation of tau, (Figure 4p), when compared to NDC and sAD astrocytes (Figure 4n,o,r).

### 3.3. Tau-Induced EAAT-2 over Expression and Deregulation of ERKs in Sporadic AD-Derived Astrocytes

**Figure 5:**
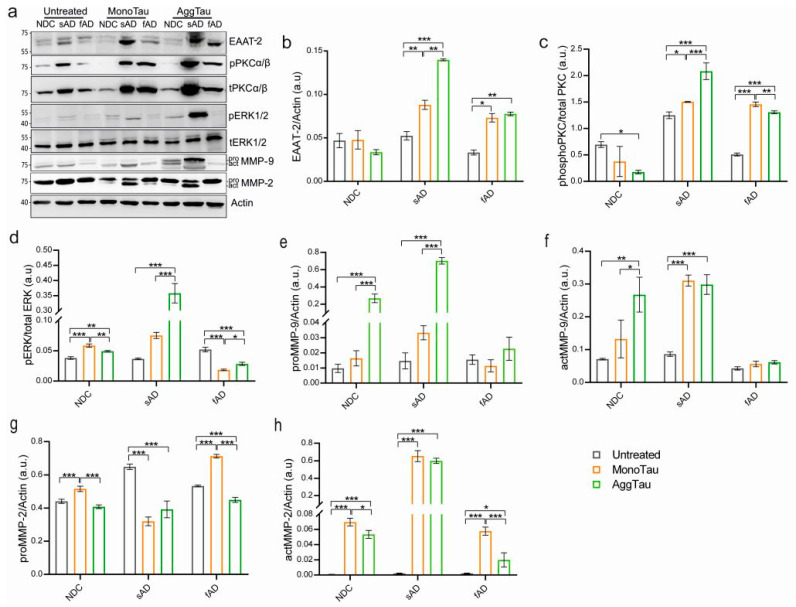
Deregulation of astrocytic protein upon exposure to tau. (**a**) Immunoblots using antibody against proteins EAAT-2, phosphoPKC II (pPKCα/β), total PKC II (tPKCα/β), phosphoERK1/2 (pERK1/2), total ERK1/2 (tERK1/2), pro- and active MMP-9 and MMP-2. Actin was used as loading control. Graph showing levels of (**b**) EAAT-2, (**c**) phospho/total PKCα/β, (**d**) phospho/total ERK1/2, (**e**) pro-MMP9, (**f**) active-MMP9, (**g**) pro-MMP2, and (**h**) active-MMP2 in astrocytes between the untreated and monomeric and aggregated tau-treated groups. One-way ANOVA followed by Tukey’s test for multiple analysis was used for statistical analysis (*n* = 3/group; *n* = 3 experimental repeats), * *p* < 0.05, ** *p* < 0.01, and *** *p* < 0.001.

In order to address the response of the astrocytes to misfolded protein tau (Mono- and AggTau), we isolated cells lysates and subjected them to immunoblotting. It was shown that under pathological conditions, EAAT-2 was present within NFTs [45], and the upregulation of EAAT-2 attributed to mutant tau expression was recently reported in Tg4510 mice [46]. Thus, we were curious to know whether exposure to tau protein (Mono- or AggTau) affects the expression levels of this transporter. Upon exposure to tau, we observed an upregulation of EAAT-2 in AD-derived clones (Figure 5a); however, the increase was ~3 fold more pronounced in sAD astrocytes upon exposure to AggTau when compared to MonoTau (Figure 5a,b). The fAD astrocytes showed similar levels of EAAT-2 upregulation in the presence of both forms of tau. These results point to the increased susceptibility of AD-derived astrocytes to pathological tau forms when compared to NDC astrocytes, which are more resistant to tau-induced changes.

Changes in astroglial EAAT are regulated by protein kinase C (PKC) [40]. Hence, we assesses whether the tau protein induced change in EAAT is PKC dependent. Using antibodies against phospho- and total PKC we observed no change in levels in NDC astrocytes upon tau (Mono- and AggTau) exposure, when compared to untreated cells (Figure 5a). In contrast, both sAD and fAD astrocytes showed increased phosphorylation of PKC upon exposure to tau proteins. The sAD astrocytes showed ~2-fold and ~1-fold higher levels of phospho-PKC upon exposure to AggTau when compared to NDC and fAD astrocytes, respectively (Figure 5c). On the other hand, the levels of phospho-PKC in fAD astrocytes were similar upon Mono- and AggTau exposure.

In AD, the hyperphosphorylation of extracellular signal-regulated kinases (ERK) is associated with NFT formation [47]. Astrogliosis in sporadic AD is linked to the activation of the ERK signaling cascade [48]. Therefore, we studied whether tau protein can induce astrocytic ERK signaling cascade in AD. For this purpose, astrocytes from NDC, sAD and fAD were stimulated with 4 µM monomeric or 1 µM aggregated tau. After 24 h, cells were lysed and analyzed for phosphorylated ERK levels using Western blot (Figure 5a). We did not observe any difference in total ERK levels between the untreated groups (Figure 5a). On the contrary, both Mono- and AggTau (Figure 5a,d) induced the phosphorylation of ERK in NDC astrocytes when compared to untreated cells (Figure 5e). We also observed 3-fold higher levels of phosphorylation of ERK in sAD astrocytes upon AggTau exposure when compared to MonoTau stimulation (Figure 5d). On the other hand, the ERK activity of fAD astrocytes decreased significantly after exposure to both Mono- and AggTau when compared to the untreated cells (Figure 5d).

### 3.4. Tau Protein Selectively Alters the Levels of Astrocytic MMP-9 and MMP-2 In Vitro

Astrocytes are the primary source of matrix metalloproteinases (MMPs), proteases that are involved in the remodeling of the brain’s extracellular matrix. It is reported that astrocytic MMPs, MMP-2 and MMP-9, are involved in the degradation and clearance of Aβ [12]. A recent study also show that MMP-2 can cleave recombinant tau in vitro [13]. However, it is unknown if iPSC-derived astrocytes can catabolize tau in vitro, and if AD-derived astrocytes differ in their ability to cleave misfolded protein tau. Using immunoblotting, we detected MMP-9 and MMP-2 in astrocyte cell lysates from all clones. We found that tau treatment (Mono- and AggTau) increased the levels of both pro- and active MMP-9 forms in astrocytes from NDC and sAD (Figure 5a). The highest levels of active MMP-9 upon MonoTau treatment were observed in sAD astrocytes (2-fold higher when compared to NDC and fAD), while AggTau induced the highest expression in both NDC and sAD clones, mainly in sAD, which showed 4-fold higher levels when compared to NDC, and manifold higher than fAD astrocytes (Figure 5f). Levels of pro- and active forms of MMP-9 in fAD astrocytes were consistently lower in all cases (Figure 5e,f). Untreated astrocytes from NDC, sAD and fAD only showed the intracellular pro-MMP-2 form (Figure 5a), and the basal levels were comparable between groups (Figure 5g). The active form of MMP-2 was detected after both Mono- and AggTau treatmentz in all cases, suggesting that tau can induce the production of active-MMP-2 in astrocytes. In response to MonoTau, the highest levels were found in sAD astrocytes (~9-fold higher than NDC and sAD), and lowest in fAD clone, akin to NDC astrocytes (Figure 5a,h). AggTau had a similar effect with the highest production of active MMP-2 by sAD (~10-fold higher than fAD) and lowest by fAD astrocytes, with NDC showing moderately higher levels of active MMP-2 (Figure 5h).

### 3.5. Tau Protein Alters the Secretion and Activity of MMPs in Conditioned Media

**Figure 6:**
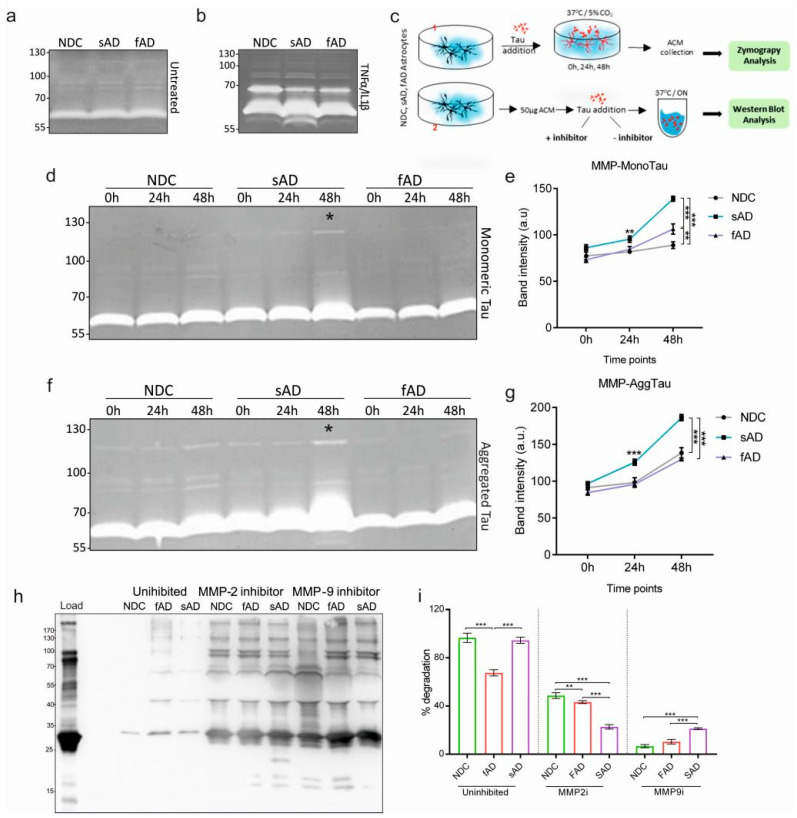
Astrocytes derived from sporadic Alzheimer’s disease iPSCs show higher gelatinase activity. (**a**) Representative zymograph image using astrocyte-conditioned media (ACM) from control, sporadic and familial iPSC-derived astrocytes. (**b**) Representative zymograph image of ACMs from control, sporadic and familial iPSC-derived astrocytes pretreated with TNFα and IL1β. (**c**) Schematics showing experimental design for the detection of MMPs and tau degradation analysis. (**d**) Representative zymograph image using ACM from control, sporadic and familial AD astrocytes treated with monomeric tau for 0 h, 24 h and 48 h. Note the moderate increase in gelatinase activity at 48 h of ACM from sAD astrocytes (asterisk). (**e**) Graphs showing difference in MMP activity after exposure to monomeric tau between the ACM from three groups. (**f**) Representative zymograph image using ACM from NDC, sporadic and familial AD astrocytes treated with aggregated tau for 0 h, 24 h and 48 h. Increase in gelatinase activity at 48 h of ACM from sAD astrocytes was observed (asterisk). (**g**) Graph showing difference in gelatinase activity of MMPs after exposure to aggregated tau between the three groups. (**h**) Degradation of aggregated tau by proteases in the ACM from three different iPSC-derived astrocytes. The protease activity is selectively inhibited by MMP2 and MMP9 inhibitors. (**i**) Graph showing percentage of tau degradation between the groups. One-way ANOVA followed by Tukey’s multiple comparisons tests, *n* = 3/group; *n* = 3 experimental repeats, * *p* < 0.05, ** *p* < 0.01, and *** *p* < 0.001.

Astrocytes constitutively secrete MMP-9 and MMP-2; however, it is unknown if AD-derived astrocytes have altered MMP secretion and activity in the presence of misfolded protein tau. Therefore, we subjected the astrocyte-conditioned medium (ACM) from all clones to gelatinase—zymography. We found gelatinase activity in all cases (Figure 6a). Pretreatment with TNF-α and IL-1β was used as a positive control to show that all cells respond to the proinflammatory stimuli (Figure 6b).

To study if misfolded tau alters MMP activity in vitro, we exposed the cells to 4 µM of monomeric tau and 1 µM aggregated tau. We collected ACM from individual clones at 0, 24 and 48 h time points and analyzed their gelatinase activity (scheme in Figure 6(c1)). We observed that the gelatinase activity of ACM increased with time upon exposure to both forms of tau species in all cases; however, the ACM from sAD astrocytes showed the highest gelatinase activity (Figure 6d,f). The degradation activity of ACM from sAD astrocytes was the highest at 48 h (asterisk in Figure 6d,f) in both cases (Figure 6e,g), and AggTau was a more potent inducer of the secretion of MMPs in sAD astrocytes when compared to MonoTau.

Since we found that tau protein can induce the secretion and activity of MMPs, specifically MMP-2 and -9, we aimed to determine if misfolded tau protein can serve as a substrate and can be processed by astrocytic MMPs. To address this question, we incubated 50 µg of ACM from the three groups with Mono- (4 µM) and AggTau (1 µM) tau for 6 h and 24 h at 37 °C (scheme in Figure 6(c2)). After incubation, samples were resolved and immunoblotted using pan-tau DC25 antibody. We did not detect any degradation of Mono- nor AggTau at the 6 h time point (Supplementary Figure S4).

At 24 h, we observed the complete degradation of monomeric tau in all cases (Supplementary Figure S4). Aggregated tau was degraded more efficiently by NDC-ACM and sAD-ACM (≥90%) (Figure 6h,i), while the degradation capacity of fAD-ACM was comparatively lower (~60–65%) than NDC- and sAD-conditioned media (Figure 6h,i). Since MMP-2 and MMP-9 are the most prominent metalloproteinases secreted by astrocytes, the ACMs were supplemented with inhibitors: i-MMP-9 and i-MMP-2. We observed that, in all cases, the addition of the inhibitors mitigated the tau degradation capacity. Although, with sAD-ACM, the levels of degraded tau were higher when MMP-9 activity was inhibited and concomitantly lower when MMP-2 activity was blocked. The predominant degrading MMP in both NDC and fAD was MMP-9, as shown by the negative activity between the inhibition of MMP-2 and MMP-9. The addition of pan-MMP inhibitor EDTA almost completely abrogated tau degradation in all NDC, sAD and fAD ACM (Supplementary Figure S4a,b).

### 3.6. Astrocyte-Conditioned Medium Leads to Upregulation of Synaptic Markers and Increased Calcium Activity of iPSC-Derived Healthy Neurons

**Figure 7:**
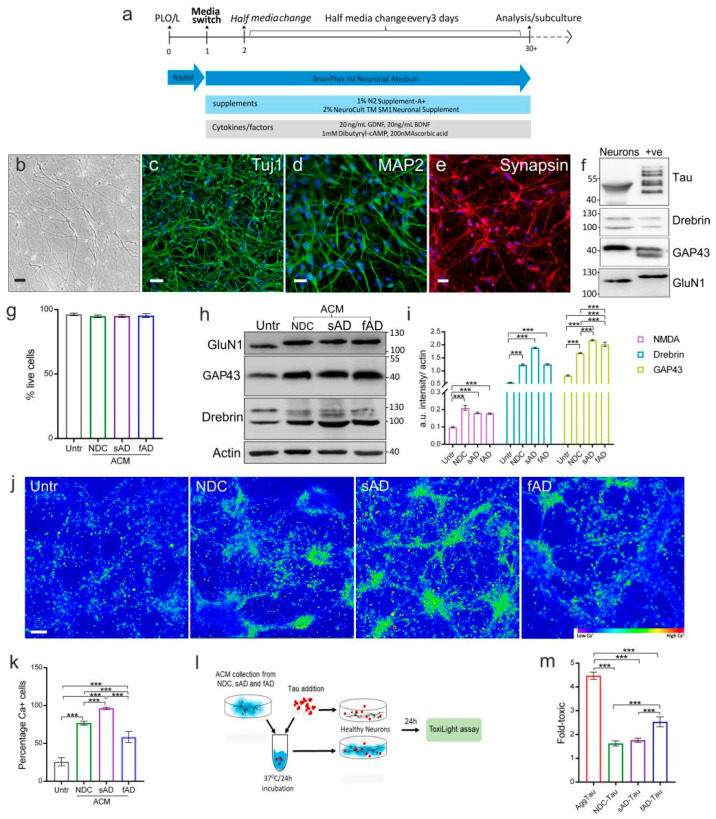
Impact of ACM on iPSC-derived healthy neurons upon glutamate stimulation. (**a**) Scheme showing differentiation of neurons from iPSC-derived NSCs. (**b**) Phase-contrast image of differentiated neurons. Scale bar: 50 μm. Representative images of neurons positive for the neuronal markers (**c**) β-tubulin III (Tuj1), (**d**) MAP2 and (**e**) Synapsin. Scale bar: (**b**,**c**) 100 μm; scale bar: (**e**) 50 μm. (**f**) Characterization of neurons by immunoblotting using antibodies against Pan-Tau DC25, and markers Drebrin, GAP43 and GluN1. Human brain lysate was used as positive control for Drebrin, GAP43 and GluN1. (**g**) Graph showing percentage of viable neurons in untreated and ACM-treated groups. (**h**) Representative immunoblots of neuronal extract after 5-day subculture with ACM supplementation from NDC, sAD and fAD astrocytes. Actin was used as loading control. (**i**) Graph showing increased levels of synaptic markers GluN1, Drebrin and plasticity marker GAP-43 in presence of ACM from NDC, sAD or fAD, when compared to untreated neurons. (**j**) Wide field images showing calcium influx in neurons upon glutamate stimulation in presence of ACM from NDC, sAD and fAD or without ACM supplement (Untr). (**k**) Graph showing percentage of Ca^2+^-positive cells (no. of Ca^2+^ cells/100 cells) upon glutamate stimulation in neurons cultured without (Untreated) or with ACM supplement. Note the highest calcium flux in sAD-ACM-supplemented neurons when compared to NDC and fAD-ACM supplementation. The percentage of calcium-positive cells from five random fields was calculated using Fiji (ImageJ 2.0.0). (**l**) Scheme showing the treatment of neurons with ACM-Tau pretreatment or tau alone and subsequent cytotoxicity analysis. (**m**) Graph showing data from toxilight assay of untreated or ACM-Tau-treated neurons. One-way ANOVA followed by Tukey’s test for multiple comparisons was used for statistical analysis, *n* = 3/group; *n* = 3 experimental repeats, *** *p* < 0.001.

Astrocytes are involved in governing vital functions of neurons, including synaptic plasticity. To study whether the secretome of iPSC-derived astrocytes has an impact on neurons, we first differentiated neurons from control iPSC-derived NSCs using a neural induction medium, maintained in a neuronal medium (Figure 7a). A microscopic examination of neurons derived from control NSCs showed numerous dendritic and axonal processes (Figure 7b). Our immunocytochemical characterization showed the presence of neuronal markers β-tubulin III (Tuj1), Microtubule-associated protein 2 (MAP2) and Synapsin I (Figure 7c–e, respectively). Moreover, using immunoblotting, we observed the presence of tau protein, Drebrin, Growth-associated protein 43 (GAP-43), and Glutamate ionotropic receptor NMDA type subunit 1 (GluN1) in cell homogenates from neurons (Figure 7f). To investigate the effect of ACM on healthy neurons, cells were cultured for one week with 300 µg ACM/clone supplement. The ACMs did not have any toxic effect on neurons (Figure 7g). The addition of ACM led to increased neurite outgrowth and branching in all cases (Supplementary Figure S5a–d), and the upregulation of the synaptic marker Drebrin, plasticity marker GAP-43, and glutamate receptor subunit, GluN1, when compared to neurons cultured without ACM (Figure 7h,i).

We next analyzed the effect of ACM on the response of neurons to glutamate stimulation using calcium imaging. For this purpose, neurons (untreated and grown with ACM supplementation) were loaded with a Fluo-4 calcium indicator and calcium transients upon glutamate stimulation were recorded (Supplementary Movies S4–S7). The stimulation of neurons with 10 nM glutamate induced calcium flux in all cases, while ACM-neuronal cultures exhibited a higher calcium influx compared to untreated neurons. The sAD ACM-treated neurons showed the highest amount of Ca^2+^-positive cells, followed by NDC neurons, and the least activity was observed in fAD-ACM cultures (Figure 7j,k). The neurons with sAD ACM showed a 2-fold higher number of cells with Ca^2+^ transients than fAD ACM neurons (Figure 7j). The numbers of cells were identical between the groups in all cases (Supplementary Figure S6).

### 3.7. Effect of ACM Treated Tau on iPSC-Derived Neurons

It is known that misfolded tau has a detrimental effect on neurons. Since we observed that ACM from individual astrocytes degraded tau protein through MMP-mediated cleavage, we studied if ACM treatment mitigates the toxic effect of misfolded tau on neurons. Therefore, we exposed neurons to aggregated tau or ACM-pretreated aggregated tau (Figure 7l). As expected, AggTau alone showed neurotoxicity; however, ACM-AggTau led to a significant reduction in toxicity for neurons in all ACM clones. The fAD-ACM/tau was still more toxic compared to NDC and sAD ACM/tau (Figure 7m).

## 4. Discussion

Recently, iPSC-derived cell models have been widely used to investigate human diseases and disorders, including AD [31,32,33,34,35]. iPSC-derived neurons from AD patients show elevated amyloid beta levels, tau hyperphosphorylation and oxidative stress [33,35,36]. In this study, we first generated and characterized iPSC-derived astrocytes from healthy non-demented (NDC), sporadic and familial Alzheimer’s disease patients. We found no morphological difference and negligible phenotypic differences between the resting astrocytes from control and AD subjects. This is in line with recently published reports showing no phenotypic difference between healthy and disease-derived astrocytes [36,49].

Cytoplasmic Ca^2+^ fluctuations in response to synaptic neurotransmitters, such as glutamate, are responsible for intercellular signaling in astrocytes [50]. Evidence on abnormal Ca^2+^ homeostasis in AD originates from rodent models, and limited information on aged human astrocytes is available [26]. Our study shows that one of the major differences in astrocytes derived from AD patients and healthy controls was their response to glutamate-induced Ca^2+^ waves. Both sAD- and fAD-derived astrocytes showed altered Ca^2+^ flux when compared to control astrocytes. Moreover, the astrocytes from sAD displayed more robust intracellular Ca^2+^ oscillations than the other groups. It was recently reported that iPSC-derived astrocytes from familial AD demonstrate altered calcium hemostasis [36]. Our results extend this observation and show that the Ca^2+^ flux in astrocytes from sporadic AD is markedly perturbed in comparison to astrocytes from NDC and fAD. Calcium deregulation is observed during aging and in the absence of dominant mutation in sAD [51]. It is proposed that AD-related aberrant glial Ca^2+^ signaling may contribute to abnormal neuronal Ca^2+^ homeostasis [52]. Indeed, we observed that the astrocyte-conditioned media from AD-derived astrocytes had a marked influence on neuronal Ca^2+^ response to glutamate stimulation. More specifically, neurons cultured with ACM from sporadic AD astrocytes showed higher levels of Ca^2+^ transients in response to glutamate, while fAD Astrocyte ACM failed to even induce glutamate-dependent neuronal Ca^2+^ transients, akin to control astrocyte ACM. This suggests that abnormal Ca^2+^ signaling in familial AD astrocytes may aggravate disease pathogenesis in AD, by deregulating neuronal activity and function.

Atrophic and reactive astrocytes are observed in brains of AD subjects [53], and the spatial localization of reactive astrocytes and NFTs correlates with the progression of the disease [53]. However, their role in the formation of pathological tau lesion is yet unknown [25]. The AD-derived astrocytes displayed changes in morphology upon tau stimulation, but astrocytes from NDC did not. In the presence of monomeric or aggregated tau, the astrocytes from AD were reactive, showing increases in cell size and morphological reshaping. This was accompanied by an increase in GFAP levels in these astrocytes, indicating a reactive state, an early phenomenon in response to stimuli. Negligible phenotypic differences or changes in GFAP levels in astrocytes from NDC were observed, despite showing tau uptake levels akin to AD-derived astrocytes. Reactive astrocytes, characterized by cellular hypertrophy and an increased expression of GFAP were observed in AD, and in proximity to pathological aggregates such as NFTs [53]. This suggests that extracellular tau protein can induce phenotypic changes in astrocytes from a resting to a reactive state. Moreover, these results also suggest that the astrocytes from AD are more prone to activation by external stimuli than astrocytes from healthy subjects.

Astrocytes from AD brain show intracellular tau accumulation [54]. We show that the astrocytes from all three groups were efficient in the uptake and degradation of monomeric tau; however, differences in the clearance of aggregated tau between the groups were observed. The fAD astrocytes were less efficient at clearing aggregated tau leading to its accumulation when compared to astrocytes from NDC and sAD. A recent study reported the incomplete digestion and accumulation of amyloid beta protofibrils by astrocytes in the lysosomes [55]. Our study demonstrates the incomplete digestion of aggregated tau and its accumulation only in fAD iPSC-derived astrocytes, and highlights a crucial difference between fAD and sAD iPSC-derived astrocytes in AD pathogenesis.

A dysregulation of astroglial ERK signaling pathways is observed in the early stages of late-onset AD [48]. ERKs are mitogen-activated kinases involved in several vital functions of cells, such as proliferation, signaling and plasticity. Hyperphosphorylated ERKs are associated with NFTs in AD cases that were mainly devoid of any amyloid deposition [47]. Therefore, the trigger for the activation of astroglial ERK in AD is yet unclear [48]. We observed hyperphosphorylation of ERKs, in response to tau protein, in astrocytes from sAD (late-onset AD), in comparison to NDC- and fAD-derived astrocytes. This shows that extracellular tau protein can signal astrogliosis and astroglial activation in sporadic AD (sAD). This activation was proposed as a compensatory mechanism for cell functionality and survival [48], suggesting that the astrocytes in sAD may delay the onset and progression of disease pathogenesis, and that this process is deregulated in fAD.

The activation of extracellular signal-regulated kinases in astrocytes plays a crucial role in inducing EAAT2 gene expression [56,57]. We observed a specific increase in the levels of EAAT-2 in AD astrocytes in response to tau. More pronounced EAAT-2 levels were observed in sAD astrocytes upon exposure to aggregated tau in comparison to fAD astrocytes. The increase in EAAT levels in astrocytes from AD in response to aggregated tau was accompanied by an increased phosphorylation of PKCα/βII and a robust increase in phospho ERK1/2 in sAD astrocytes when compared to fAD astrocytes. It is reported that PKC levels correlates with EAAT-2 levels in astrocytes, and its inhibition alters EAAT-2 expression [57]. Therefore, our result demonstrates that aggregated tau induces the upregulation of EAAT-2 expression in AD-derived astrocytes, and this process is dependent on PKC-mediated signaling pathways. On the other hand, drastic increase in phospho ERK1/2 was observed in sAD astrocytes, but their levels were reduced upon exposure to both forms of tau in fAD astrocytes. This suggests that the tau protein can activate ERK signaling cascade in sAD astrocytes, which is perturbed in fAD astrocytes.

The ERK pathway is associated with the upregulation and secretion of MMP-2 [58], and MMP-9 [59,60], and the process of MMP-9 upregulation is mediated via PKCs in astrocytes [59]. We observed that the upregulation of ERK and PKC in sAD astrocytes was accompanied by increased levels of intracellular MMP-2/9 in response to both monomeric and aggregated tau protein. The increase in the production of MMPs was also reflected in the secretome of sAD-derived astrocytes, consistent with ERK activation, which showed the highest gelatinase activity at 48 h upon exposure to monomeric and aggregated tau forms. Put together, these results show that misfolded tau augment the production and secretion of MMPs from sAD-derived astrocytes when compared to fAD astrocytes.

It is suggested that MMP-2 and MMP-9 are involved in the degradation of Aβ into non-toxic fragments and exert protective effect on neurons in AD [61]. Moreover, knock-out of both MMPs resulted in accumulation of Aβ [11,62,63]. Here, we show that astrocytic MMPs are also able to degrade tau proteins to different extent. Moreover, the sAD astrocyte ACM showed higher levels of MMP activity in response to Mono- and AggTau and concomitantly higher degradation efficiency of AggTau, when compared to fAD astrocytes in vitro. This suggests that the sAD astrocytes are more efficient in the secretion of MMPs in response to extracellular tau and promote its degradation in the brain. On the other hand, extracellular tau did not augment MMP activity in fAD astrocytes, suggesting a less effective clearance of tau, which may lead to its deposition in a diseased brain.

Astrocytes are shown to exert a neuroprotective effect on neurons [64]. The exposure of ACM from control and AD astrocytes to iPSC-derived healthy neurons did not induce any neurotoxic effect but proved to be beneficial by increasing the levels of synaptic proteins, namely GAP-43 and Drebrin. In addition, the ACM treatment of aggregated tau also alleviated its toxic effect on neurons in vitro when compared to aggregated tau-treated neurons alone. The addition of ACM from NDC, sAD and fAD astrocytes modulated the neuronal response to glutamate stimulation. We found that Ca^2+^ influx was elicited upon glutamate stimulation in neurons cultured with ACM when compared to untreated cells. The response was more augmented in neurons with sAD astrocyte-derived ACM when compared to fAD astrocytes. This was accompanied by an upregulation of GluN1, the synaptic marker Drebrin, and the synaptic plasticity marker GAP-43. Changes in GluN1 or NMDAR levels induced by synaptic plasticity and by spatial memory formation in neurons have been observed [65]. We speculate that the increased Ca^2+^ flux upon glutamate stimulation in ACM-supplemented neurons might be due to the upregulation of the NMDAR receptor and synaptic plasticity. However, the enhanced Ca^2+^ response of neurons treated with sAD-ACM may be partially independent of the NMDAR-dependent plasticity since the levels of GluN1 did not differ between the ACM clones.

Astrogliosis and ERK activation is speculated as an early response to amyloid production and deposition [48], but no astroglial ERK activation was associated with amyloid deposits in AD; however, activated ERKs were in proximity to NFTs. We observed the phosphorylation of ERK and reactive astrogliosis in response to tau. This suggests that tau released from neurons in the early stages of AD may trigger an astrocytic response, which may be neuroprotective [48]. Changes in the microenvironment of CNS, such as the accumulation of cytokines and NOS, BBB damage, etc., may alter astrocyte function, leading to the inefficient clearance of pathological tau in the late stages of Alzheimer’s disease.

A limitation in the study might be that we used heparin-induced tau aggregates, which are widely used to model AD filaments [66,67,68], and studies have used in vitro-assembled tau aggregates on glial cells [44,69]. We used human truncated tau aa151-391 (fragment present in the core of AD PHF,) which exhibits robust fibrillation and induced neurofibrillary pathology in rodent models, akin to AD [24,70]. A recent study showed that tau filaments induced using heparin are more polymorphic than filaments from AD [71]. However, heparin-induced Tau fibrils are composed of cross-β structures with parallel stacking reminiscent of AD PHFs [71]. Nevertheless, future studies should focus on addressing the effect of highly pure AD-PHFs on healthy and AD iPSC-derived astrocytes.

**Figure 8:**
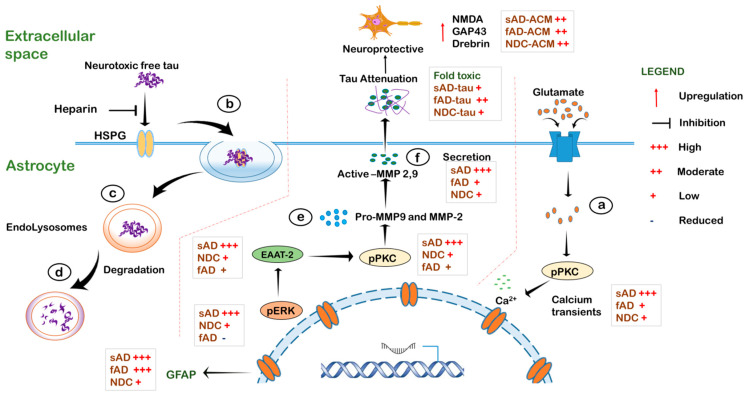
Schematic showing differential responses in the control and Alzheimer’s disease iPSC-derived astrocytes. (**a**) Astrocytes generated from Alzheimer’s disease (sAD and fAD) show increased glutamate-induced calcium transients in terms of amplitude and duration when compared to healthy astrocytes (NDC). (**b**) Extracellular free tau, which is neurotoxic, is taken up by astrocytes via heparin sulphate proteoglycan (HSPG), a process mitigated by heparin. Upon tau exposure, only AD astrocytes exhibit GFAP upregulation and morphological reshaping, attributes of reactive state. Tau is processed in the endo-lysosomal compartment (**c**) and degraded (**d**). Meanwhile, the uptake of tau activates ERK pathway with highest levels of pERK, EAAT-2 and pPKC in sporadic AD-derived astrocytes (sAD), leading to the upregulation of astrocytic pro-MMP-2 and MMP-9 (**e**), which results in differential activation and secretion (**f**). The capacity to degrade and attenuate extracellular tau was the highest when using the secretome of sAD and NDC astrocytes compared to fAD, and showed a neuroprotective effect accompanied by an increase in the levels of neuronal NMDA, GAP43 and drebrin.

One of the key observations in our study is that the astrocytes from sporadic AD differ from familial AD astrocytes. Some differences in patient iPSC-derived neurons from sporadic and familial AD have already been reported [72]. For example, the neurons derived from fAD were more responsive to Aβ1-42 oligomers when compared to neurons from sAD [73]. We observed that, despite showing morphological similarities, GFAP levels, altered calcium activity, and the ability to uptake tau, the astrocytes from sAD demonstrated higher EAAT-2, pPKC, pERK and active MMP-9 and MMP-2 levels when compared to astrocytes from fAD (Figure 8). The sAD astrocytes also demonstrated higher gelatinase activity in response to monomeric and aggregated tau. Moreover, the ACM from sAD showed a robust clearance of aggregated tau and was more neuroprotective than fAD ACM/tau. The ACM from sAD augmented the Ca^2+^ response of neurons upon glutamate stimulation when compared to fAD ACM. Overall, this suggests that the astrocytes from sporadic AD demonstrate a more robust and enhanced activity than astrocytes from familial AD.

## 5. Conclusions

In conclusion, a major difference between familial and sporadic Alzheimer’s disease is the time of onset. Not only were the fAD-derived astrocytes inefficient in the degradation/clearance of tau protein, but the secretome from fAD was also less efficient in clearing extracellular tau. Since sporadic AD is characterized by a late onset, as opposed to early familial AD progression, a set of compensatory mechanisms are possibly involved in the delay of the disease. Our study provides evidence that sporadic AD-derived astrocytes are hyperactive in response to pathological tau protein via the activation of ERK and PKC signaling pathways, which are shown to be neuroprotective. Our study highlights differences in healthy, sporadic and familial AD astrocytes and the influence of different forms of tau protein on Alzheimer’s disease-derived astrocytes. Future studies should focus on the cytokine profile and signaling molecules of diseased astrocytes post tau stimulation to elucidate its effect on their functions, as well as its impact on neurons and ultimately CNS homeostasis.

## Data Availability

All results that lead to the conclusion of the study are in the manuscript or in Supplementary Material. The datasets of the study are available from the corresponding authors upon reasonable request.

## Supplementary Materials

The following are available online at https://www.mdpi.com/article/10.3390/cells11091429/s1: Supplementary Figure S1: Characterization of iPSC-derived neuronal stem cells. Figure S2: Spontaneous calcium transients in iPSC-derived astrocytes. Figure S3. Blocking of HSPG with heparin mitigates the effect of tau proteins on sAD and fAD iPSC-derived astrocytes. Figure S4: Degradation of tau at 6 h by astrocytic MMPs. Figure S5: Neurons from healthy NDCs with or without conditioned medium from NDC, sAD and fAD astrocytes. Figure S6: Representative images of neurons from healthy NDCs with or without conditioned medium from NDC, sAD and fAD astrocytes after glutamate stimulation. Supplementary Table S1: Clones used in the study. Movie S1: NDC_Astrocytes_Glutamate stimulation.mov. Movie S2: sAD_Astrocytes_Glutamate stimulation.mov. Movie S3: fAD_Astrocytes_Glutamate stimulation.mov. Movie S4: Neurons_Untreated_Glutamate stimulation.mov. Movie S5: Neurons_NDC-ACM_Glutamate stimulation.mov. Movie S6: Neurons_sAD-ACM_Glutamate stimulation.mov. Movie S7: Neurons_fAD-ACM_Glutamate stimulation.mov.
