# Supplementary material for: Astrocytes Derived from Familial and Sporadic Alzheimer’s Disease iPSCs Show Altered Calcium Signaling and Respond Differently to Misfolded Protein Tau
Source: Cells. 2022 Apr 22;11(9):1429. doi: 10.3390/cells11091429 (PMC9101114; doi:10.3390/cells11091429)
Supplement: Supplementary file 1 [file cells-11-01429-s001.zip › revised Supplementary_Material_1632644.pdf]

Supplementary Materials for

Astrocytes derived from familial and sporadic Alzheimer's disease iPSCs show altered calcium signaling and respond differently to misfolded protein tau

Veronika Brezovakova[1], Eva Sykova[1], Santosh Jadhav[1]*

Correspondence to: santosh.jadhav@savba.sk

This file includes:

Figures. S1 to S6

Table. S1.

Other Supplementary Materials (as attachment) for this manuscript include the following:

Movies M1 to M7(.mov)

Supplementary figures

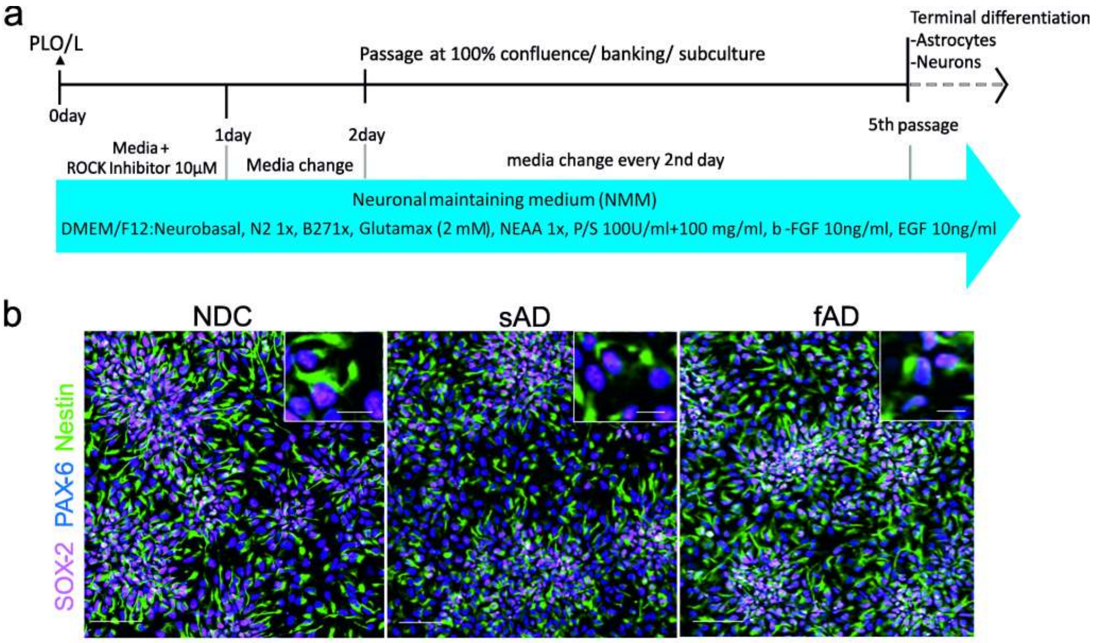

Figure. S1. Characterization of iPSC derived neuronal stem cells. (**a**) Schematic overview of the culture and cryo-banking of iPSC derived neuronal stem cells. (**b**) Immunocytochemical analysis of neuronal stem cells from NDC, sAD, and fAD using stem cells antibodies SOX-2, PAX-6 and Nestin. Highly pure NSCs uniformly expressing the markers were observed in all cases. Scale bar: 100 µm; inset: 20 µm.

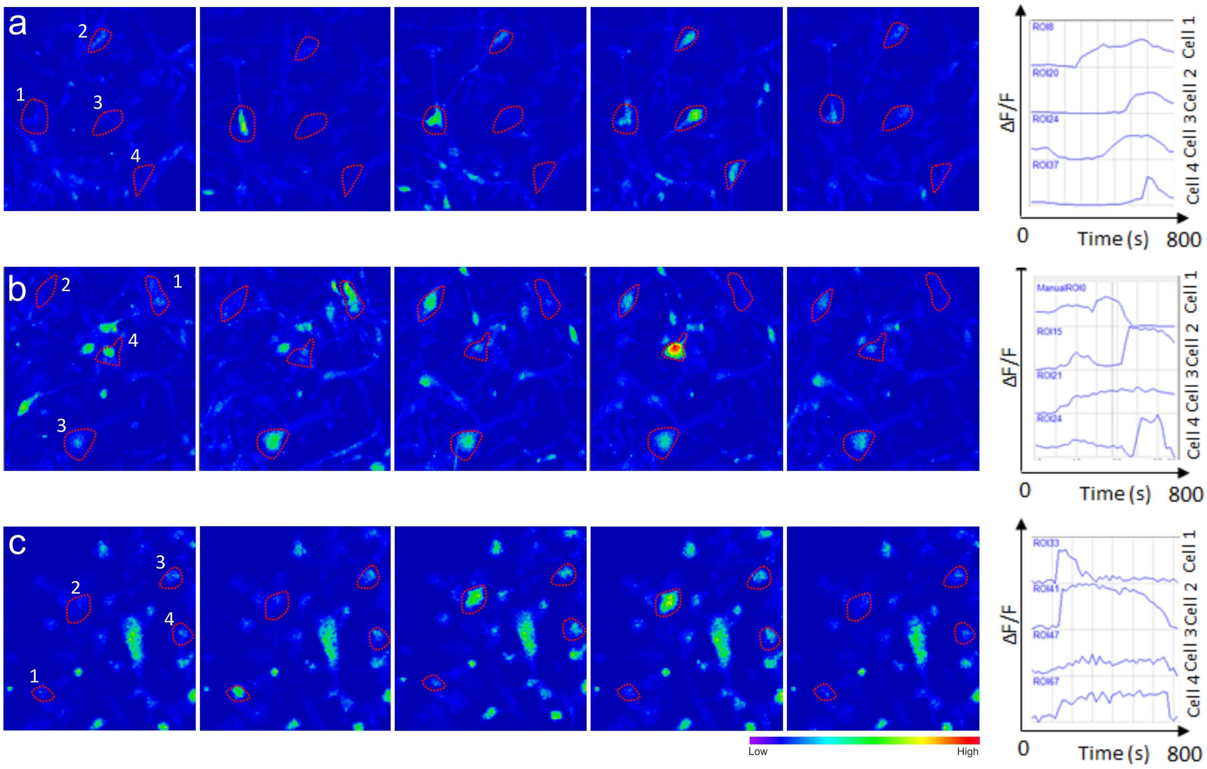

Figure. S2. Spontaneous calcium transients in iPSC derived astrocytes. Representative images of **c**alcium transients from (**a**) non-demented controls, (**b**) sporadic AD, and (**c**) familial AD are shown. Individual cells from each clones are marked (red) and labelled (numbers), and their calcium flux peaks generated using CALIMA are shown. n=3/ group; n=3 experimental repeats.

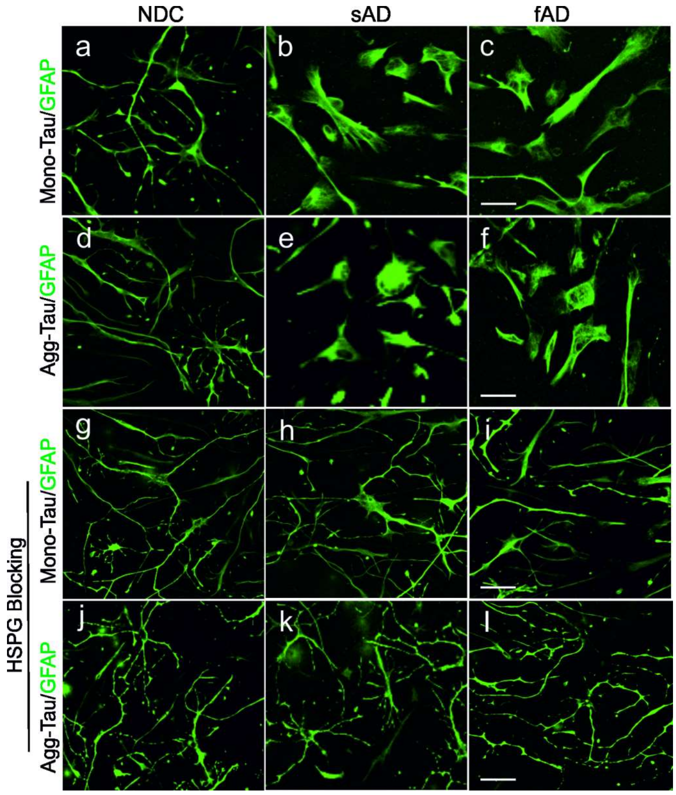

Figure. S3. Blocking of HSPG with heparin mitigates the effect of tau proteins on sAD and fAD iPSC derived astrocytes. Representative confocal images using anti-GFAP antibody on NDC, sAD and fAD derived astrocytes with (g-l) or without heparin (a-f) pre-incubation (20 µg/ml for 3 h) and tau treatment. The mono- and aggtau induced hypertrophy on sAD and fAD astrocytes was mitigated by heparin.

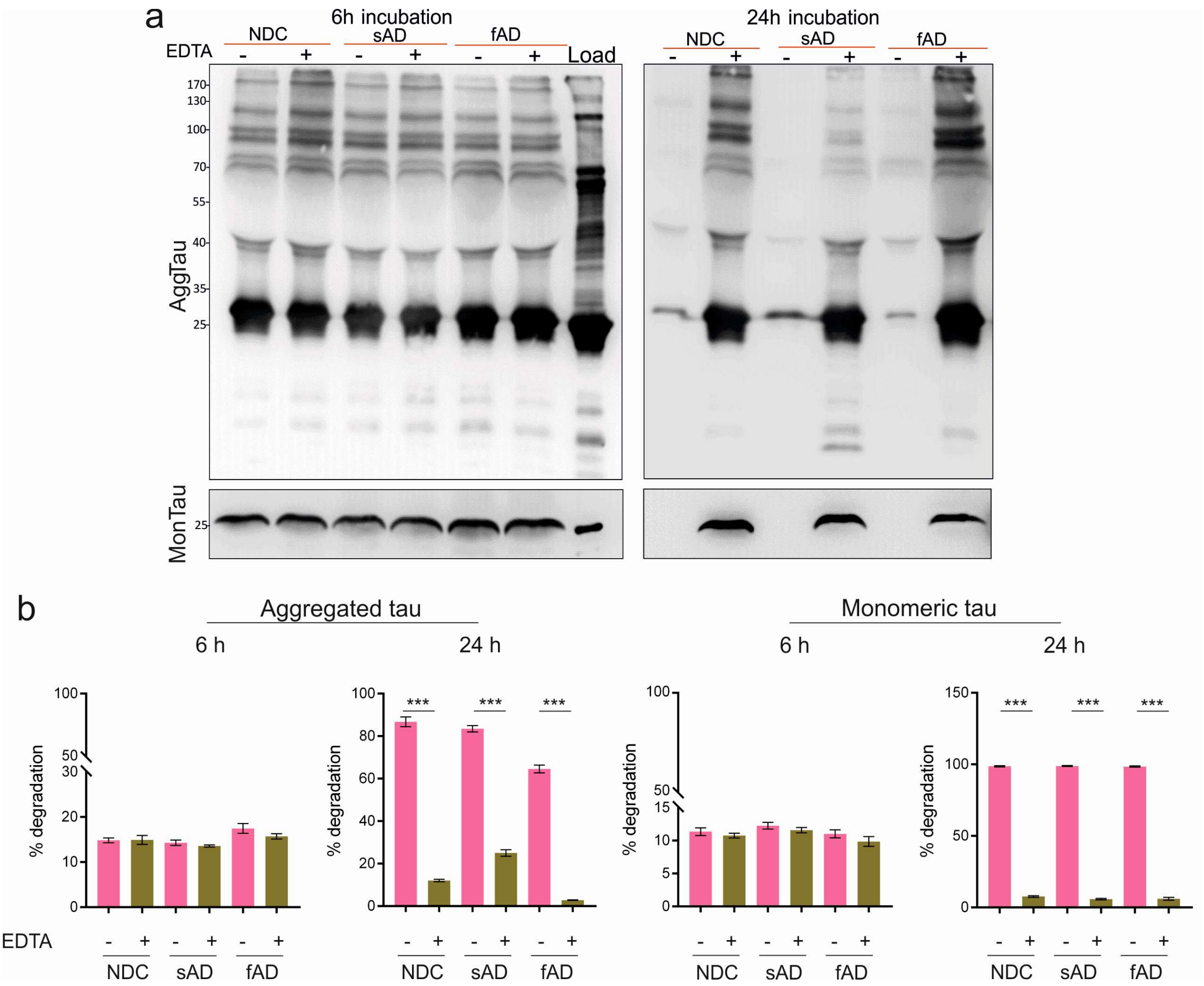

Figure. S4. Degradation of tau by astrocytic MMPs. (a) Immunoblotting using pan-Tau antibody DC25 to assess Tau degradation by ACM from healthy, sAD and fAD at 6h and 24 h respectively. Top panel show degradation of aggregated tau (Agg-Tau), and bottom panel show degradation of monomeric tau (Mono-Tau). Universal MMP inhibitor EDTA was used to suppress degradation activity (+) and ACM without EDTA was used as controls (-). Respective load of proteins is shown for each panel. **(b)** Graphs showing percentage degradation of aggregated tau and monomeric tau at 6 h and 24 h respectively. No degradation of both tau forms was observed at 6 h but only after 24 h time point. EDTA inhibited the degradation activity of MMPs in all cases. Mann Whitney test was used to compare differences between the EDTA treated and untreated group (n=3), ***<0.0001.

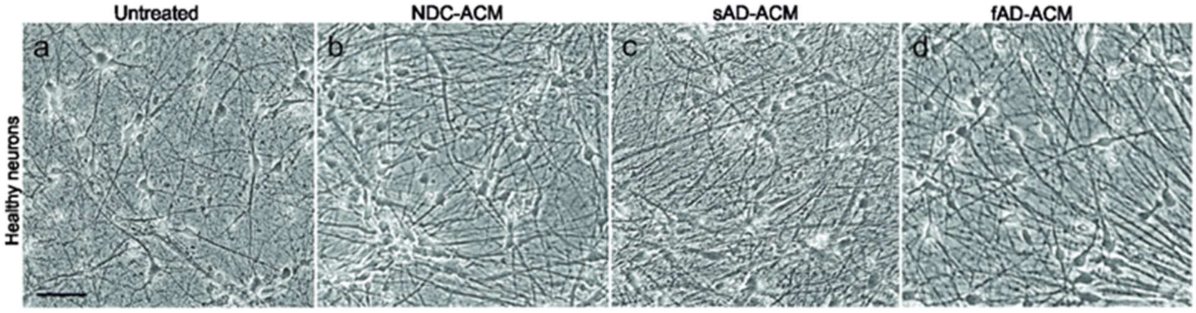

Figure. S5. Neurons from healthy NDCs with or without conditioned medium from NDC, sAD and fAD astrocytes. Bright-field images of neurons differentiated from iPSCs from healthy control (**a**) without ACM, or with ACM from (**b**) NDC astrocytes, (**c**) sAD astrocytes and (**d**) fAD astrocytes. Scale bar: 100 µm.

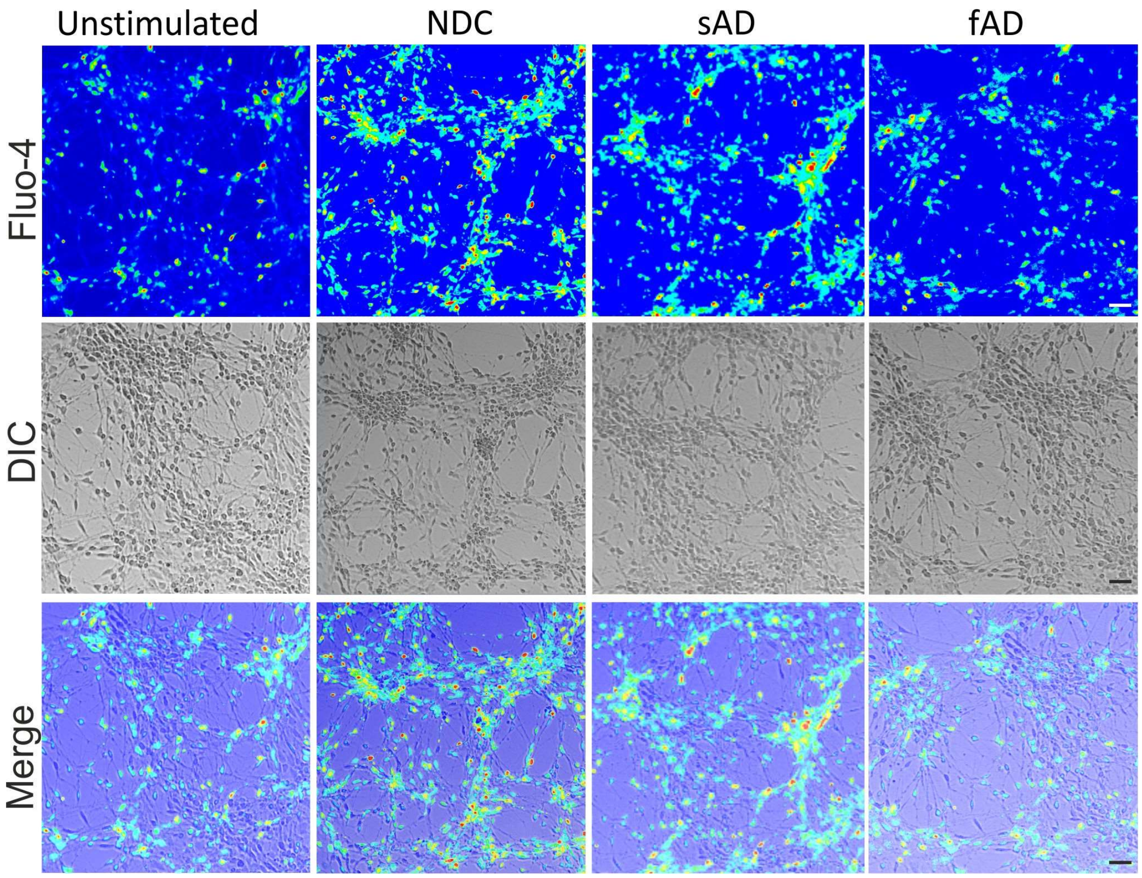

Figure. S6. Representative images of Neurons from healthy NDCs with or without conditioned medium from NDC, sAD and fAD astrocytes after glutamate stimulation. Images showing Fluor-4 signal (Ca^{2+} transients), Bright-field (DIC) and merged signals. Note that there is no noticeable difference in the number of cells between the groups. Scale bar: 50 µm.

Supplementary tables:

Table S1. Clones used in the study

**Clones**	**Gender**	**Age during biopsy**	**Starting material**	**Mutation**
NDC	Female	35	PBMCs	-
NDC	Female	> 70	PBMCs	-
NDC	Female	NA	PBMCs	-
Sporadic AD*	Female	57	PBMCs	-
Sporadic AD*	Female	75	PBMCs	-
Sporadic AD*	Female	63	PBMCs	-
Familial AD	Female	55	PBMCs	PSEN1 (V89L)
Familial AD	Female	31	Fibroblast	PSEN1 (A246E)
Familial AD	Female	NA	Fibroblast	PSEN1 (M146L)

* Genetic predispositions in sporadic cases (if any) are unknown, NA: Not available.

List of movies.
M1_NDC_Astrocytes_Glutamate stimulation.mov
M2_sAD_Astrocytes_Glutamate stimulation.mov
M3_fAD_Astrocytes_Glutamate stimulation.mov
M4_Neurons_Untreated_Glutamate stimulation.mov
M5_Neurons_NDC-ACM_Glutamate stimulation.mov
M6_Neurons_sAD-ACM_Glutamate stimulation.mov
M7_Neurons_fAD-ACM_Glutamate stimulation.mov
